# Réglementation et régulation : Exemple de l'Agence Ivoirienne de régulation de la mutualité sociale en Côte d'Ivoire

**DOI:** 10.48327/mtsimagazine.n1.2021.95

**Published:** 2021-04-28

**Authors:** J.-V. Ayité

**Affiliations:** Directeur Général, Programme d'Appui aux Stratégies Sociales (PASS)

**Keywords:** Mutualité, Mutualiste, Mutuelle, Côte d'Ivoire, Régulation, Protection sociale, Mutuality, Mutualist, Mutual insurance, Côte d'Ivoire, Regulation, Social protection

## Abstract

L'Agence ivoirienne de régulation de la mutualité sociale (AIRMS) est l'organe administratif de la mutualité sociale en Côte d'Ivoire. Elle a pour mission d'instruire les demandes d'agrément des mutuelles sociales et de leurs structures faîtières, des les immatriculer et de les contrôler.

Dans la pratique, l'AIRMS s'est positionnée comme un véritable partenaire du mouvement mutualiste ivoirien. Privilégiant une approche par la sensibilisation, l'agence a encouragé les mutuelles à se mettre aux normes édictées par le règlement communautaire de la mutualité sociale de la zone UEMOA afin de devenir des acteurs crédibles dans le paysage du financement de la santé en Côte d'Ivoire.

Par ailleurs, la Directrice Générale de l'Agence, Mme Koné Collette, est à l'avant-garde du plaidoyer pour l’évolution de la participation des femmes dans la gouvernance des structures mutualistes. Elle porte avec le Programme d'appui aux Stratégies sociales (PASS) un ambitieux programme dans ce sens: le Réseau International des Femmes Solidaires (RIFES).

## Abstract (en)

The Agence ivoirienne de régulation de la mutualité sociale (AIRMS) is the administrative body for social mutuality in Côte d'Ivoire. Its mission is to examine, register and monitor applications for approval of social mutual insurances and their umbrella organizations.

In practice, AIRMS has positioned itself as a true partner of the Ivorian mutualist movement. Favoring an awareness-raising approach, the agency has encouraged mutual insurances to meet the standards set by the community regulation of social mutuality in the WAEMU zone to become credible players in the health financing landscape in Côte d'Ivoire. In addition, the Director General of the Agency, Ms. Koné Collette, is at the forefront of advocacy for the evolution of women's participation in the governance of mutualist structures. With the Programme d'appui aux Stratégies sociales (PASS), she is leading an ambitious program in this regard: the Réseau International des Femmes Solidaires (RIFES).

La zone de l'Union Économique et Monétaire Ouest Africaine (UEMOA) est la première zone dans le monde à s’être dotée d'une réglementation commune encadrant la mutualité. De la création de la mutuelle à la cessation de ses activités en passant par les modalités de gouvernance, de gestion, ou son développement, ce règlement a pour objet de s'assurer que les mutuelles soient des institutions crédibles et de protéger les populations qui y adhèrent. L'UEMOA fait ainsi obligation à chaque état membre dans la région de mettre en place une agence de régulation de la mutualité sociale ayant pour mission de faire appliquer les recommandations du règlement communautaire.

L'article 23 du Règlement N°07/2009/CM/UEMOA portant réglementation de la mutualité sociale au sein de la zone de l'Union économique et monétaire ouest africaine (UEMOA), exige que chaque Etat membre se dote d'un organe administratif de la mutualité sociale ainsi qu'un registre national d'immatriculation des mutuelles sociales. La Côte d'Ivoire a été l'un des premiers pays à se doter de cet organe administratif.

L'agence ivoirienne de régulation de la mutualité sociale (AIRMS) a été créée par décret n°2012-588 du 27 juin 2012. Elle a pour mission de réglementer la vie mutualiste dans le pays à travers, l'instruction des dossiers d'agrément des mutuelles sociales et la tenue du registre nationale d'immatriculation des mutuelles sociales. Sous tutelle du ministère de l'Emploi et de la Protection Sociale, l'AIRMS a également pour attribution le contrôle pour le bon fonctionnement des mutuelles avec les ratios de gestion édicté par le règlement communautaires, mais elle a aussi la responsabilité de prendre toutes les mesures conservatoires nécessaires, à la sauvegarde des intérêts des membres des mutuelles

Dès les débuts, l'AIRMS a dû faire face au défi de sa structuration et de son mécanisme de travail, en tant que premier modèle dans la zone. Composé de services clés comme la direction de l'agrément et de l'immatriculation, la direction du suivi et contrôle, la direction administrative et financière et le service de communication, l'AIRMS a réussi à se doter d'un plan d'action afin d'atteindre ses objectifs assignés dans le cadre de sa mission.

Dans la pratique, l'AIRMS s'est positionné comme un partenaire du mouvement mutualiste ivoirien. La politique mise en place par cette structure dès le début de ses activités a été d'encourager les mutuelles à se mettre aux normes de l'UEMOA afin de devenir des acteurs crédibles dans le paysage du financement de la santé en Côte d'Ivoire. Elle s'est focalisée sur la diffusion d'information, la formation et la sensibilisation pour aider et orienter les mutuelles et les acteurs de la mutualité.

L'AIRMS, c'est aujourd'hui plus d'une quarantaine de mutuelles de santé immatriculées. Cette « certification » de l'activité de certaines mutuelles leur a plaider en leur faveur au moment où l’état devait sélectionner les premiers organismes de gestion déléguée de la Couverture sanitaire universelle mise en place dans le pays. L'agence contribue au quotidien à apporter des solutions aux contentieux qui émergent au sein des mutuelles.

L'AIRMS fédère également les actions des mutuelles sur certains sujets. Dans les activités de plaidoyer notamment, l'AIRMS veille à renforcer le positionnement des mutuelles dans le paysage sanitaire du pays, mais aussi, dans l'organisation de la réponse mutualiste aux crises sanitaires. En nommant une femme à la tête de cette agence, l’État de Côte d'ivoire envoyait déjà un signal fort au monde mutualiste. Cela est d'autant plus significatif qu'en Afrique, les femmes ont en effet une approche très pragmatique dans la mise en place et l'animation des actions solidaires. Elles y jouent un rôle important. Malheureusement, la proportion des femmes occupant des postes de responsabilités dans les organisations du secteur de la santé solidaire reste encore faible. Aujourd'hui, la Directrice Générale de l'Agence, Mme Koné Collette, est à l'avant-garde du plaidoyer pour l’évolution de la participation des femmes dans la gouvernance des structures mutualistes. Elle porte avec le Programme d'appui aux Stratégies social Pass un ambitieux programme dans ce sens.

Après plusieurs années d'opération, la Directrice générale de l'agence à su faire accepter cette institution dans le paysage mutualiste local et régional et positionner l'AIRMS comme une agence de référence en la matière à la disposition des agences sœurs du Niger et du Mali pour partager son expérience et participer à la construction d'une mutualité plus forte et plus solidaire dans la région.

## Conflits D'intérêts

Les auteurs ne déclarent aucun conflit d'intérêts.

